# Topographical aspects in the dynamics of sleep homeostasis in young men: individual patterns

**DOI:** 10.1186/1471-2202-12-84

**Published:** 2011-08-16

**Authors:** Thomas Rusterholz, Peter Achermann

**Affiliations:** 1 Institute of Pharmacology and Toxicology, University of Zurich, Winterthurerstrasse 190, 8057 Zurich, Switzerland; 2 Neuroscience Center Zurich, University and ETH Zurich, Winterthurerstrasse 190, 8057 Zurich, Switzerland; 3 Zurich Center for Integrative Human Physiology, University of Zurich, Winterthurerstrasse 190, 8057 Zurich, Switzerland

## Abstract

**Background:**

Sleep homeostasis refers to the increase of sleep pressure during waking and the decrease of sleep intensity during sleep. Electroencephalography (EEG) slow-wave activity (*SWA*; EEG power in the 0.75-4.5 Hz range) is a marker of non-rapid eye movement (NREM) sleep intensity and can be used to model sleep homeostasis (Process *S*). *SWA *shows a frontal predominance, and its increase after sleep deprivation is most pronounced in frontal areas. The question arises whether the dynamics of the homeostatic Process *S *also show regional specificity. Furthermore, the spatial distribution of *SWA *is characteristic for an individual and may reflect traits of functional anatomy. The aim of the current study was to quantify inter-individual variation in the parameters of Process *S *and investigate their spatial distribution. Polysomnographic recordings obtained with 27 EEG derivations of a baseline night of sleep and a recovery night of sleep after 40 h of sustained wakefulness were analyzed. Eight healthy young subjects participated in this study. Process *S *was modeled by a saturating exponential function during wakefulness and an exponential decline during sleep. Empirical mean *SWA *per NREM sleep episode at episode midpoint served for parameter estimation at each derivation. Time constants were restricted to a physiologically meaningful range.

**Results:**

For both, the buildup and decline of Process *S*, significant topographic differences were observed: The decline and buildup of Process *S *were slowest in fronto-central areas while the fastest dynamics were observed in parieto-occipital (decrease) and frontal (buildup) areas. Each individual showed distinct spatial patterns in the parameters of Process *S *and the parameters differed significantly between individuals.

**Conclusions:**

For the first time, topographical aspects of the buildup of Process S were quantified. Our data provide an additional indication of regional differences in sleep homeostasis and support the notion of local aspects of sleep regulation.

## Background

Three distinct processes underlie sleep regulation (for a recent review see [1]). A homeostatic process, reflecting prior history of sleep and waking, plays a major role in sleep regulation. Additionally a circadian process, a clock-like mechanism independent of sleep and wake history, modulates sleep. Furthermore, an ultradian process occurs within sleep, reflected by the cyclic alternation of non-rapid-eye-movement (NREM) sleep and rapid-eye-movement (REM) sleep. The circadian and homeostatic processes were formalized in the two-process model of sleep regulation [2,3]. According to this model, a homeostatic process (Process *S*) and a circadian process (Process *C*) interact to generate sleep-wake patterns. Slow-wave activity (*SWA*; EEG power in the 0.75 - 4.5 Hz range) serves as a marker for sleep homeostasis and thus for modeling of Process *S*. *SWA *shows a decline in the course of sleep that can be approximated by an exponential decrease across NREM sleep episodes [2-6]. The level of *SWA *in the first NREM sleep episode is dependent on the duration of prior waking and is best described with a saturating exponential function [2,3,7,8]. Thus, Process *S *is characterized by an exponential decline during sleep towards a lower asymptote (*LA*) and a saturating exponential increase towards an upper asymptote (*UA*) during wakefulness.

Process *S *was originally assumed to be a global process and the parameters of Process *S *were mainly derived from central or fronto-central derivations (see overview in [6]). Bos et al. [9], assuming that the EEG is the outcome of a stochastic process with similar or identical behavior over wide cortical areas, proposed that delta activity during sleep measured at different EEG derivations has a common source, but different gain factors and independent noise sources. Similarly, Merica and Fortune [10] argued that sleep structure (NREM-REM sleep cycles) results from brainstem mechanisms, whereas derivation specific aspects result from cortico-cortical interactions. However, *SWA *shows a frontal predominance and its percentagewise increase after sleep deprivation is most prominent in frontal areas [11-17]. Further evidence for regional and use dependent differences in the sleep EEG was obtained by experimental manipulation of specific brain regions during waking and the resulting local changes in the corresponding areas during subsequent sleep [18-20]. Such observations raise the question of whether the dynamics of Process *S *show regional specificity. Our data base with 27 EEG derivations covering the scalp is well suited to address this question. Since sleep homeostasis is mainly reflected in the time constants, this paper focuses on the spatial distribution of the time constants. Furthermore, we examine the spatial distribution of the lower and upper asymptotes. Their absolute values reflect *SWA *in an individual and exhibit age-dependent changes [21-27]. In our previous publication [6] we hypothesized that the distance between the normalized asymptotes (*UA - LA*) might reflect the capacity of the brain to generate slow waves. Thus, we expect that the distance between the asymptotes will also show regional differences.

We hypothesize that we will find regional differences in our measures of sleep regulation which reflect plastic brain processes occurring during wakefulness and sleep as articulated in the synaptic homeostasis hypothesis of Tononi and Cirelli [28,29].

Based on an elaborated model [30], Zavada et al. [31] investigated regional aspects of *SWA *regulation in recordings based on 26 EEG derivations. In this model, the decline of Process *S *was assumed to be proportional to the momentary level of *SWA*. Zavada et al. [31] reported a higher efficiency in *SWA *dissipation in frontal derivations, a finding reflecting local aspects in sleep regulation. However, they only examined the decline of the homeostatic process during baseline sleep.

We recently established a robust method for parameter estimation of Process *S *on an individual basis and observed considerable inter-individual variation, particularly in the time constants [6]. In this paper we investigate the spatial distribution of the parameters of Process *S *within individuals by applying this new approach. The data base consisted of EEG data of 27 derivations recorded during baseline sleep and during sleep following 40 h sleep deprivation. Because the spatial distribution of *SWA *is characteristic for an individual [32] and may reflect traits of functional anatomy, we also quantified inter-individual variation in the parameters of Process *S*.

## Results

### Parameters derived from normalized averaged data

**Figure 1:**
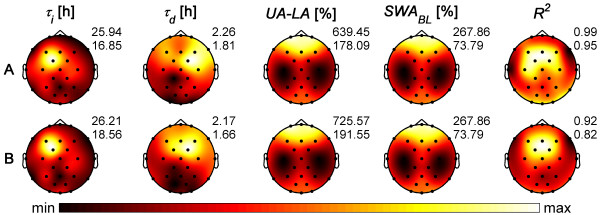
**Topographical maps of the parameters of Process *S***. *(A) *Parameters estimated for average normalized data (see Methods for details of normalization). *(B) *Average of the parameters derived for each participant separately. Maps of individuals are illustrated in Figure 2. Left to right: Increasing time constant *τ_i_*, decreasing time constant *τ_d_*, distance between upper and lower asymptote (*UA-LA*), mean *SWA *in NREM sleep (stages 2-4) of the first 7 h 27 min of baseline sleep (*SWA_BL_*), and goodness of fit *R^2^*. Each map was scaled separately between minimal and maximal values. At the top right of the maps, maxima (max) and minima (min) of the maps are indicated.

The homeostatic Process *S *was modeled by a decreasing exponential function during sleep (parameters: time constant of decline *τ_d_*, lower asymptote *LA*; see equation 1 in Methods) and a saturating exponential function during waking (parameters: time constant of buildup *τ_i_*, upper asymptote *UA*; see equation 2 in Methods). The parameters of Process *S *were first estimated for average data across subjects in each of the 27 derivations (see Methods). The corresponding topographical maps are illustrated in Figure 1A. In addition, the spatial distribution of *SWA *for baseline sleep and the goodness of fit are displayed. The time constants of the increase, *τ_i_*, varied between 16.9 and 25.9 h (Table 1; Figure 1A). The largest value, corresponding to the slowest buildup of Process *S*, was found at the fronto-central derivation FC1 (Table 2) whereas the fastest buildup was observed at F7 (left frontal; Table 2). The time constants of the decline, *τ_d_*, were in the range of 1.8 to 2.3 h (Table 1; Figure 1A) with the slowest decrease (largest time constant) of Process *S *at the frontal-central derivation FC2 (Table 2) and the fastest decrease at the occipital derivation O1 (Table 2). In general, a slower dynamic of Process *S *was present in fronto-central areas compared to other regions.

**Table 1:** Parameters derived from normalized averaged data across participants

	*τ_i _*[h]	*τ_d _*[h]	*UA-LA *[%]
range	16.85 - 25.94	1.81 - 2.26	178.09 - 639.45
mean	19.41	2.01	373.68
STD	2.14	0.12	120.00
CV	0.11	0.06	0.32

Range, mean value, standard deviation (STD) and coefficient of variation (CV; STD/mean) across the 27 derivations are shown. *τ_i_*, increasing time constant; *τ_d_*, decreasing time constant; *UA-LA*, distance between upper and lower asymptote.

**Table 2:** Electrode positions of maxima and minima in the maps of the parameters of Process *S*

	*τ_i_*			*τ_d_*			*UA-LA*		
	min	max	p-value	min	max	p-value	min	max	p-value
A	F7	FC1	6.06E-05	O1	FC2	3.94E-04	C3	Fp1	2.12E-03
B	F8	FC1	2.03E-04	PO1	FC2	9.14E-04	C3	Fp1	2.12E-03

*τ_i_, τ_d _*time constants of increase and decrease, and *UA-LA *distance between upper and lower asymptote. Maxima (max) and minima (min) position of each parameter were localized in maps derived from parameter estimation in normalized average data over participants (A, see maps in Figure 1A) and maps of mean parameters estimated with normalized individual data (B, see maps in Figure 1B). To assess topographical differences, estimated parameters in individuals at the corresponding location of the maxima and minima were compared by paired t-test (p-value). Electrode positions are indicated in Figure 6.

The distance between the upper and lower asymptotes (*UA-LA*) showed a similar topographical pattern as *SWA *in baseline and recovery sleep, i.e. a frontal predominance, as well as the increase of *SWA *from baseline to recovery sleep with the most prominent increase in frontal areas (Figure 1A, only *SWA *of baseline sleep is shown).

The goodness of fit was generally high (*R^2 ^*> 0.95, adjusted coefficient of determination) as expected for average data. The best fit was observed in fronto-central areas whereas the worst fit occurred in temporal areas (Figure 1A).

### Parameter distribution in individuals

**Figure 2:**
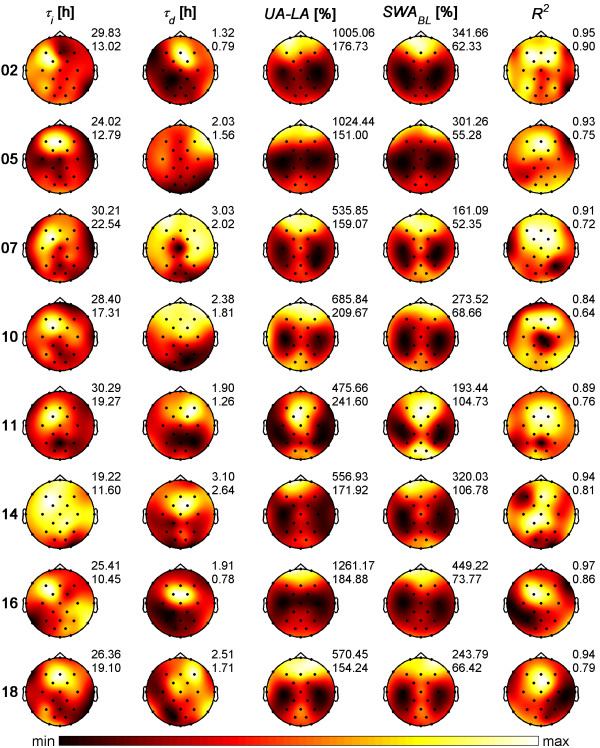
**Topographical maps of the parameters of Process *S *in eight individuals**. Estimations were based on normalized data (see Methods). Left to right: Increasing time constant *τ_i_*, decreasing time constant *τ_d_*, distance between upper and lower asymptote (*UA-LA*), mean *SWA *in NREM sleep (stages 2-4) of the first 7 h 27 min of baseline sleep (*SWA_BL_*), and goodness of fit *R^2^*. Each map was scaled separately between minimal and maximal values. At the top right of the maps, maxima (max) and minima (min) of the maps are indicated.

Next, the parameters of Process *S *were estimated for each individual separately. Figure 2 shows the maps of the parameters estimated from normalized individual data. Although distinct individual spatial patterns were observed, the decline and buildup of Process *S *were slowest in fronto-central areas across participants. The fastest decreases occurred mainly in parieto-occipital areas whereas the fastest increases could not be assigned to a specific area.

The goodness of fit between simulations and empirical data was generally largest in fronto-central areas (Figure 2). Only one subject showed the largest goodness of fit in the centro-parietal region (CP1, subject 14). The location of the smallest goodness of fit varied between individuals and occurred primarily in temporal, parietal and occipital areas.

**Figure 3:**
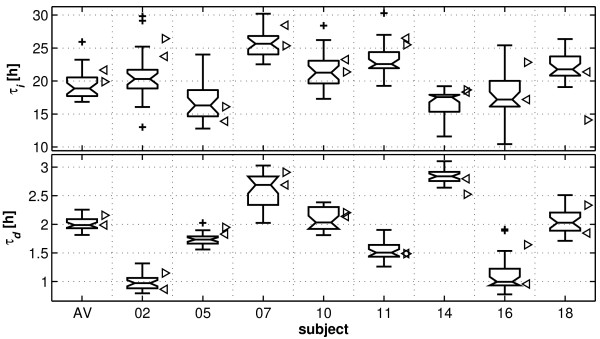
**Distribution of the time constants across derivations**. Boxplots of the time constants (*τ_i_*, increase; *τ_d_*, decrease) across derivations in the average map (AV; Figure 1A) and in individual maps (Figure 2). In addition, time constants estimated for derivation C3 against average reference and C3A2 [6] are plotted to the right of each boxplot (left and right oriented triangle, respectively). Boxes represent the lower quartile, median, and upper quartile. The notch indicates the 95% confidence interval of the median. Maximum whisker length is 1.5 times of the inter-quartile range. They extend to the most extreme data value that is not an outlier. Outliers are indicated by +.

The variation of the time constants within and between individuals is depicted in the boxplots of Figure 3. Non-overlapping notch areas between the different boxplots point to significant differences between individuals. To assess the influence of the reference on the time constants, time constants estimated for derivation C3 against average reference and C3A2 [6] are also indicated in Figure 3 (left and right oriented triangle, respectively). In most individuals the time constants of C3 determined with respect to these two references differed considerably but did not reach statistical significance.

Maps averaged across individuals closely resembled those derived from average data (Figure 1B) with value ranges of *τ_i _*and *UA-LA *slightly larger and values of *τ_d _*slightly smaller than those for average data. Mean of goodness of fit showed a spatial distribution similar to average data but with lower values.

**Figure 4:**
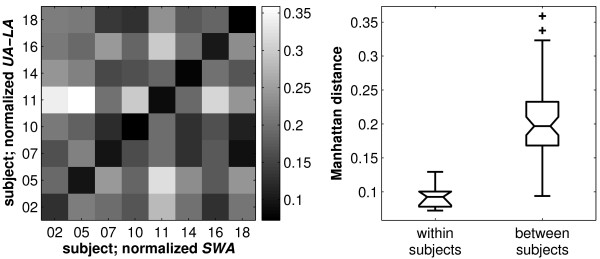
**Comparison of individual normalized maps of the distance between asymptotes (*UA-LA*) and *SWA *in baseline sleep**. *Left: *Differences (Manhattan distances; see Methods) between all pairs of maps plotted as a gray-scale coded matrix. Smallest distances were present within individuals (diagonal). Numbers denote individuals. *Right: *Distribution (boxplot) of within (N = 8) and between (N = 56) subject differences (Manhattan distance). Within subject differences were significantly lower than between subject differences (notch areas do not overlap). For details about the representation of boxplots see Figure 3.

Similarly, maps of the distance between upper and lower asymptotes (*UA-LA*) were comparable to the *SWA *patterns (Figure 2). However, the correspondence was less striking than those observed in the patterns of average data (Figure 1). As maps of *SWA *are characteristic for an individual [32] and may reflect traits of the underlying functional anatomy, we compared maps of the distance between upper and lower asymptotes with *SWA *maps to determine whether the same spatial pattern was exhibited in both. To compare these maps, the Manhattan distance (*MD*) between normalized maps of *UA-LA *and *SWA *were examined. The calculated *MD*s were generally smaller within individuals than between individuals (Figure 4), except for subject 18, suggesting to a larger similarity of the maps within individuals. Most importantly, the within-subjects distribution of *MDs *differed significantly from the between-subjects distribution (Boxplots in Figure 4 right; notch areas did not overlap).

### Topographical differences in the dynamics of Process *S*

Topographical differences in the parameters of Process *S *(*τ_i_, τ_d _*and *UA-LA*) were assessed by comparing the individual parameters at the location of maxima and minima determined in the maps of average data and in the mean maps of individually estimated parameters (Figure 1). Table 2 lists the corresponding electrode positions at which maxima and minima were observed. Parameter values at the position of maxima and minima differed significantly (paired t-test; one test for each parameter; Table 2) pointing to regional differences in the dynamics of Process *S*.

Interestingly, maxima derived from average data and the mean of individual parameters were found at the same position (FC1 for *τ_i_*, FC2 for *τ_d _*and FP1 for *UA-LA*) but not the minima of the time constants (F7, F8 for *τ_i_*; O1, PO1 for *τ_d_*, respectively). The minima in the distance between the asymptotes (*UA-LA*) were invariably observed at C3.

## Discussion

This was the first study analyzing topographical differences in the parameters of Process *S *of the two-process model of sleep regulation [2,3]. Parameter estimation of Process *S *was based on a recently established method [6] which also allowed for parameter estimation in individuals. Also Zavada et al. [31] analyzed topographical aspects of sleep homeostasis. However, they only focused on the decline of Process *S*. For the first time, spatial aspects in the buildup of Process *S *were investigated, which also allowed us to investigate differences between the asymptotes (*UA-LA*) and their relation to *SWA*.

Significant topographic differences in the parameters of Process *S *were observed for both the decrease and the buildup of the homeostatic Process *S*. Distinct individual patterns in the spatial distribution of the parameters were observed (Figure 2). These differences in the dynamics of Process *S *contradict earlier views that postulated that *SWA *measured at different derivations has a common source but different gain factors [9,10]. Merica and Fortune [10] investigated correlations between power in various frequency bands of a few derivations within single NREM sleep episodes. They observed high correlations between derivations in all NREM sleep episodes. However, such high correlations between derivations do not allow to conclude that the homeostatic process would show the same dynamics in all derivations.

Homeostasis is mainly reflected in the time constants, i.e. in the speed of the buildup and decay of the homeostatic process. The faster the buildup (shorter time constant), the quicker the upper asymptote is reached; this suggests that brain areas differ in their susceptibility to sleep deprivation. The faster the decay (shorter time constant), the quicker the lower asymptote is reached which indicates that brain regions differ in the speed of dissipation of sleep pressure. *SWA *showed a frontal predominance (Figure 1) and its increase after sleep deprivation was most pronounced in frontal areas. One might have expected that the time constants of Process *S *show an inverse spatial distribution to *SWA*, with a fast (small values) increase and decline of Process *S *in frontal areas where *SWA *is highest. However, both the increase and decline of *S *were slowest in fronto-central areas. The interpretation of these findings is difficult. One hypothesis that remains to be tested could be that the differences in the dynamics of sleep homeostasis are related to the functional specialization of different cortical regions. For example, frontal lobes are thought to be responsible for higher cognitive function. As cognitive processing is essential for human functioning, it might be conceivable that these areas require a slower dynamic to minimize their vulnerability to challenges. Thus, it remains to be determined whether the speed of the deterioration of higher cognitive function during sleep deprivation is reflected in the time constants of the buildup (*τ_i_*) in frontal areas.

The synaptic homeostasis hypothesis as proposed by Tononi and Cirelli [28,29] implies that the role of sleep is synaptic downscaling: this process reduces the synaptic strength that is high at the beginning of sleep due to plastic processes occurring during wakefulness. Similar to sleep homeostasis based on the EEG, the dynamics of synaptic homeostasis may show comparable differences in the corresponding spatial patterns. However, it has been postulated that synaptic potentiation is also related to space requirements [29]. In a recent paper we hypothesized (for a discussion see [6]), that the asymptotes might reflect the capacity of the brain to generate or produce slow waves. In this paper we observed that the spatial distribution of the distance between upper and lower asymptote (*UA-LA*) closely resembled the spatial distribution of *SWA *(Figures 2 and 4). This finding supports our previous hypothesis. Then, if one assumes differences in available space for synapse formation during potentiation, it may be conceivable that the distance between the asymptotes reflects such an aspect resulting in brain region specific differences in the capacity to generate slow waves.

For both, the buildup and decline, distinct spatial patterns in the parameters of Process *S *were observed which may be characteristic for an individual similar to the spatial distribution of power in different frequency bands [32] and may reflect traits of the underlying functional anatomy. Because the topography of *SWA *and the distance between the asymptotes showed very similar patterns and the topography of *SWA *is an individual fingerprint [32], we hypothesize that the distance between the asymptotes also represents an individual fingerprint. Whether this holds also for the spatial distribution of the time constants is an open question. To assess whether time constants within an individual are stable over time, experiments with repeated sleep deprivation within an individual are required. We analyzed whether the dynamics of sleep homeostasis, in particular the time constants, have trait-like aspects using existing data of a repeated sleep deprivation protocol [33]. Parameter estimation based on a single derivation indicated that the homeostatic Process *S *is a trait [34].

A number of studies have shown that the spatial distribution of *SWA* changes with age. For example, Kurth et al. [24] demonstrated that cortical maturation occurs in a region dependent manner and is reflected in *SWA*, i.e. the location of maximal *SWA* undergoes a shift from posterior to anterior regions with increasing age. Furthermore, Robillard et al. [27] showed an age dependent reduction in the initial value of Process *S* at sleep onset primarily in anterior regions of men which was more widespread in women. The prominent increase in *SWA* in frontal brain areas due to prolonged wakefulness also appears to be age-dependent [26]. Because we observed a close relationship between the topography of the distance between upper and lower asymptote and the spatial distribution of *SWA*, we hypothesize that the distance between the asymptotes will also reflect developmental and age related alterations of brain morphology.

**Figure 5:**
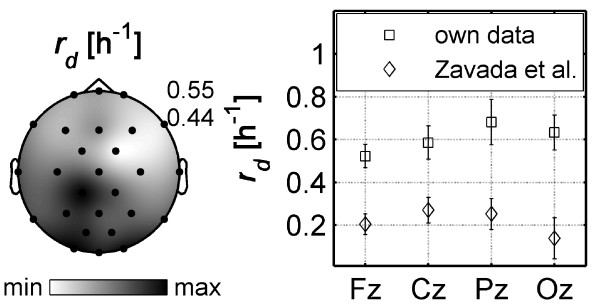
**Decay rate and comparison with data from Zavada et al. **[31]. *Left: *Topographical map of the decay rate (*r_d _= 1/τ_d_*) estimated for average data (inverse of 2^nd ^map in Figure 1A). *Right: *Decay rate of four midline derivations along the anterior-posterior axis. Own data (mean ± standard error) are compared with data of Zavada et al. [31].

Zavada et al. [31] also investigated regional aspects of sleep homeostasis based on an extended model of ultradian *SWA *regulation [30]. In the extended model, the decay of Process *S *is proportional to the momentary level of *SWA*. Additionally, *S *has a permanent rise term. The gain constant, *gc*, is used to determine the speed of the decline of *S*. Thus, *gc *can be thought of as being approximately proportional to the inverse of the time constant of the decline (i.e. the decay rate, *r_d _= 1/τ_d_*). To facilitate comparison with the maps of Zavada et al. [31], we plotted the decay rate *r_d _*as a grey scale map (Figure 5, left). In the study of Zavada et al. [31] the topographical distribution of *gc *in different frequency bands was larger over frontal regions while our data revealed an occipital focus (Figure 5, left). Zavada et al. [31] determined the decay based on baseline sleep and did not estimate the buildup of the homeostatic process based on data of sleep deprivation which may contribute to the disparate findings. Furthermore, Zavada and colleagues kept the buildup fixed and equal in all individuals. In our case, both, the buildup and the decay were estimated simultaneously based on baseline and recovery sleep after prolonged wakefulness. Given the large inter-individual differences observed in the dynamics of sleep homeostasis, the discrepancy between our data and the study of Zavada et al. [31] may simply be a consequence of inter-individual variation as different cohorts were investigated.

Furthermore, Zavada et al. [31] estimated the decay rates in the 2-3 Hz range along the anterio-posterior axis (Fz, Cz, Pz and Oz) by fitting exponential functions. In Figure 5 (right) we compared their decay rates with our own data. The distribution of the decay rates along the anterio-posterior axis was similar, however, our decay rates were larger. These differences in the absolute values of the decay rate may result from the different frequency ranges analyzed (2-3 Hz vs. 0.75-4.5 Hz).

Zavada et al. [31] reported greatest incidence of simulation failures over occipital areas. In our approach, the quality of fit (*R^2^*) was best in fronto-central areas and worst in temporal areas. This indicates that not all derivations can be fitted equally well. However, parameter estimation converged in all individuals and at all derivations. It is important to note that we limited time constants to a physiological meaningful range [6].

Zavada et al. [31] proposed a distinction between a global Process *S *related to the timing of sleep and waking which interacts with Process *C *and a local homeostatic process related to *SWA *regulation, which they called Process *Z*. Based on their results of topographic differences in *SWA *regulation, they concluded that Process *S *and *Z *are separate processes. Thus far there is no data to support a local cortical manifestation of Process *C*. The interaction of a constant Process *C *with a regional different Process *Z *would result in a regional different timing of sleep and waking. Therefore, such dissociation between a global Process *S *and a local Process *Z *seems reasonable at first, if we accept a global timing of sleep and waking. Recent data, however, point to heterogeneity in the process of sleep onset in the range of several minutes in different cortical regions [35]. This finding may question the assumption of a global timing of sleep and waking. Furthermore, it was recently shown, that most slow waves and their underlying active and inactive neuronal states occurred locally and not globally [36,37].

It is difficult to reach a final conclusion regarding the significance of the observed topographic differences of the parameters of Process *S*. However, these regional differences in the parameters may be related to plastic brain processes occurring during wakefulness and sleep (synaptic homeostasis [28,29]) which may vary across brain regions. If we accept that a global homeostatic process exists (Zavada's conjecture), the question arises how to measure and quantify the dynamics of such a global process, i.e. how to estimate its parameters. Furthermore, time constants derived from C3A2 and C3 against average reference differed considerably (Figure 3). Thus, caution is necessary when comparing parameters derived from different derivations or from the same derivation with different references.

One limitation of our study is that only male subjects were investigated which limits generalization of our findings. Future directions would be to include women. Furthermore, the small sample size (n = 8) may not allow us to capture the full range of inter-individual variation in the topography of sleep homeostasis. Nonetheless, the overall pattern of regional differences in sleep regulation was consistent across participants despite significant interindividual variability.

## Conclusions

Based on our recently established method, which allows for the estimation of the parameters of Process *S *on an individual basis, we were able to investigate regional aspects of sleep homeostasis. We found significant topographical differences in the dynamics of Process *S*, for both, its buildup and decline. In addition, individuals showed distinct spatial patterns in the parameters of Process *S*.

## Methods

### Participants & study procedure

A dataset of a previous study was analyzed [12,32]. Eight healthy young men (21-25 years, mean age 23 years) without sleep disturbances participated in the study. They were right handed, non-smokers and reported moderate alcohol and caffeine consumption. Subjects were recruited based on a questionnaire about their sleep habits that revealed regular bedtimes from approximately 23:00 to 07:00 h. Furthermore, a screening night served to exclude sleep disturbances such as sleep apnea, nocturnal myoclonus, prolonged sleep latency (> 30 min) and low sleep efficiency (< 80%).

**Figure 6:**
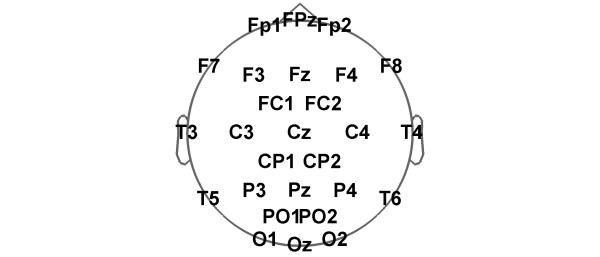
**Schematic diagram of the 27 scalp electrode positions in an extended 10-20 system**.

Polysomnographic data were recorded during an adaptation night, a subsequent baseline night and a recovery night after 40 hours of sustained wakefulness. Bedtime was scheduled at 23:00 h with sleep limited to 8 h in the adaptation and baseline nights and to 12 hours in the recovery night. During baseline and recovery sleep 27 scalp EEG electrodes (extended 10-20 system; Figure 6) were recorded. During sleep deprivation, subjects were under constant supervision by a member of the research team. During the experimental sessions and during the 3 preceding days, subjects were required to abstain from alcohol and caffeine consumption. Furthermore, they had to adhere to regular bedtimes (23:00 to 07:00 h) for 3 days prior to the study verified by ambulatory activity monitoring.

The study protocol was approved by the institutional ethical committee and participants signed informed consent.

### Data analyses

Sleep stages were scored for 20-s epochs according to Rechtschaffen and Kales [38]. For topographical analysis, EEG derivations were re-referenced to the average reference. For each derivation, power density spectra were calculated for consecutive 4-s epochs (FFT, Hanning window, linear detrending, no overlap). Sleep stages were matched with corresponding consecutive five 4-s epochs of power density spectra. *SWA *was defined as power in the frequency range of 0.75 to 4.5 Hz. Artifacts were excluded visually on a 4-s basis by inspection of derivation C3A2 and semi-automatically in the 27 EEG derivations (for details see [32]). NREM and REM sleep episodes were determined according to Feinberg and Floyd [39]. Neither skipped first REM sleep episodes nor sleep onset REM sleep episodes were present in our recordings.

### Modeling sleep homeostasis

The homeostatic Process *S *was modeled by a decreasing exponential function during sleep and a saturating exponential function during waking.

(1) $$S\left(t\right)=\left(S_{SO}-LA\right)*\text{exp}\left(-\frac{t}{\tau_{d}}\right)+LA\;\text{during}\;\text{sleep}$$

(2) $$S\left(t\right)=\left(S_{WU}-UA\right)*\text{exp}\left(-\frac{t}{\tau_{i}}\right)+UA\;\text{during}\;\text{waking}$$

where *S_SO _*is the level of *S *at sleep onset, *S_WU _*is the level of *S *at the time of wake up, *LA *is the lower asymptote, *UA *is the upper asymptote, *τ_d _*is the time constant of the decreasing exponential function and *τ_i _*is the time constant of the increasing exponential function. Time *t *starts with zero at sleep onset and at wake up, respectively.

Parameter estimation was performed as described in Rusterholz et al. [6]. The main features of the procedure are as follows. Mean *SWA *per NREM sleep episode (stages 2 to 4) at episode midpoints served as empirical data for least-squares parameter estimation. Data were normalized within each subject with mean *SWA *across all derivations in the first 7 h 27 min of baseline sleep (minimal duration of sleep in all participants). Therefore, *SWA, UA, LA *and the distance between the asymptotes (*UA-LA*) are expressed in percentage. To achieve a steady state situation of Process *S *at sleep onset during normal nights, estimations were not solely based on sleep data from a baseline and recovery day (sleep and waking periods), but on 4 sleep data sets by repeating single days: baseline, baseline (the single baseline data were repeated), sleep deprivation, recovery sleep, followed again by the baseline data in the second night after recovery (see Figure 1 in Rusterholz et al. [6]).

Parameter estimation was first performed with average data across participants (4 NREM sleep episodes in baseline and 5 episodes in recovery sleep; number of episodes common to all subjects). For the sleep-wake timing, sleep onset time and sleep lengths were averaged for baseline and recovery sleep. For parameter estimation within an individual, time constants of each derivation were limited to a physiologically meaningful range defined as values near the time constant estimated for average data (± 20 h for *τ_i _*and ± 2 h for *τ_d _*[6]). Furthermore, the *LA *was forced to be larger or equal zero. All NREM sleep episodes and individual sleep length were used for parameter estimation of individual participants. Five optimization runs with different random initial parameters in a given range were performed in an attempt to avoid local minima. Parameters of the output with the smallest error were used as optimal results.

Parameters were estimated for all 27 derivations. Maps of the parameters were plotted with the function 'topoplot' of EEGLAB [40].

### Statistics

The goodness of fit between empirical data (repeated baselines not included) and simulations was assessed by the adjusted coefficient of determination *R*^2^.

(3) $$R^{2}=1-\left(1-r^{2}\right)\frac{n-1}{n-p-1}\;\text{with}\;r^{2}=1-\frac{SS_{err}}{SS_{tot}}$$

where *SS_err _*is the residual sum of squares; *SS_tot _*the total sum of squares between observations and mean value; *n *the number of data points; and *p *the number of regressors. This approach adjusts for the number of regressors used and the different number of data points of individuals [6]. Fisher's z-transform was applied to average *R^2 ^*values of individuals.

Topographical differences in the parameters of Process *S *were assessed with paired t-tests by comparing individual parameters at the derivation where the maximum occurred with the ones where the minimum was observed (one test for each map). Maxima and minima were determined in the maps of the average data (Figure 1A) and in the mean maps of individually estimated parameters (Figure 1B).

Furthermore, topographical maps of *UA-LA *and *SWA *looked quite similar within an individual. To evaluate the affinity between maps of *UA-LA *and *SWA*, the Manhattan distance (*MD*; L1 norm) between the normalized maps was calculated within and between subjects. Each map was normalized by the sum across all derivations (proportional scaling; range 0 to 1).

(4) $$MD=\underset{i}{\sum }|{\left(UA-LA\right)}_{i}-SWA_{i}|$$

where *MD *is the Manhattan distance across all derivations *i, UA-LA *is the normalized distance of the asymptotes and *SWA *is the normalized *SWA *in NREM sleep of the first 7 h 27 min of baseline sleep. Thus, *MD *results in a value between zero and two.

## Authors' contributions

TR participated in the data analysis, interpretation of the results and preparation of the manuscript. PA participated in the design of the study, data collection, interpretation of the results and preparation of the manuscript. Both authors approved the final version of the manuscript.
